# *Mycobacterium tuberculosis* PE_PGRS41 Enhances the Intracellular Survival of *M. smegmatis* within Macrophages Via Blocking Innate Immunity and Inhibition of Host Defense

**DOI:** 10.1038/srep46716

**Published:** 2017-04-25

**Authors:** Wanyan Deng, Quanxin Long, Jie Zeng, Ping Li, Wenmin Yang, Xinchun Chen, Jianping Xie

**Affiliations:** 1 State Key Laboratory Breeding Base of Eco-Environment and Bio-Resource of the Three Gorges Area, Key Laboratory of Eco-environments in Three Gorges Reservoir Region, Ministry of Education, School of Life Sciences, Institute of Modern Biopharmaceuticals, Southwest University, Chongqing, China; 2 Key Laboratory of Molecular Biology for Infectious Diseases (Ministry of Education), Institute for Viral Hepatitis, Department of Infectious Diseases, The Second Affiliated Hospital, Chongqing Medical University, Chongqing, PR China; 3 Department of Pathogen Biology, Shenzhen University School of Medicine, 3688, Naihai Bvld, Shenzhen 518060, China

## Abstract

The success of *Mycobacterium tuberculosis (M. tuberculosis*) as a pathogen is largely contributes to its ability to manipulate the host immune responses. The genome of *M. tuberculosis* encodes multiple immune-modulatory proteins, including several members of the multi-genic PE_PPE family. Despite of intense research, the roles of PE_PGRS proteins in mycobacterial pathogenesis remain elusive. The function of *M. tuberculosis* PE_PGRS41, characterized by an extended and unique C-terminal domain, was studied. Expression of PE_PGRS41 in *Mycobacterium smegmatis*, a non-pathogenic species intrinsically deficient of PE_PGRS, severely impaired the resistance of the recombinant to multiple stresses via altering the cell wall integrity. Macrophages infected by *M. smegmatis* harboring PE_PGRS41 decreased the production of TNF-α, IL-1β and IL-6. In addition, PE_PGRS41 boosted the survival of *M. smegmatis* within macrophage accompanied with enhanced cytotoxic cell death through inhibiting the cell apoptosis and autophagy. Taken together, these results implicate that PE_PGRS41 is a virulence factor of *M. tuberculosis* and sufficient to confer pathogenic properties to *M. smegmatis*.

Tuberculosis (TB), caused by *Mycobacterium tuberculosis*, remains a global burden of morbidity and mortality. There were 10.4 million new cases and 1.4 million deaths reported in 2015 by the World Health Organization (www.whoint/tb/publications/global_report/). *M. tuberculosis* survives and persists in the host by modulating host immune responses. Upon infection, *M. tuberculosis* cell envelope-associated factors interact with the innate immune cells^1^, to tip the balance between the host and pathogen^2^. The deciphering of the *M. tuberculosis* genome uncovers a unique multigenic PE_PGRS proteins with a highly conserved PE domain and a variable PGRS domain^3^. PE is named after N-terminal Pro(P)-Glu(E) sequence, and PGRS means polymorphic GC-rich repetitive sequence. PE_PGRS proteins restrict to virulent mycobacteria^4^, such as *M. tuberculosis, Mycobacterium bovis, Mycobacterium marinum, Mycobacterium ulcerans* and *Mycobacterium kansasii*. Mycobacterial PE_PGRS are cell surface proteins implicated in virulence^5^,^6^ and pathogen-host interaction^5^,^7^.

To further elucidate the role of PE_PGRS family proteins in molecular pathogenesis of tuberculosis, we generated and characterized a prototypical member of *M. tuberculosis* PE_PGRS family protein PE_PGRS41, also known as acid and phagosome regulated protein C (aprC). PE_PGRS41 modulates pH-driven adaptation to the macrophage phagosome^8^. We investigated whether *M. tuberculosis* PE_PGRS41 could modulate signaling pathways of the host innate immune system and sought to identify crucial host target molecules involved in such interaction. *M. smegmatis* expressing PE_PGRS41 affected macrophage activation, and significantly decreased the expression and production of TNF-α, IL-1β and IL-6 by comparison to control strains. We found that LPS priming of macrophages immediately prior to infection with PE_PGRS41 expressing *M. smegmatis*, enhances TNF-α, IL-1β and IL-6 mRNA levels and secretion. Furthermore, *M. tuberculosis* PE_PGRS41 blocked innate defense response against Mycobacteria by decreasing cell apoptosis and autophagy and increasing cell death of macrophage, resulted in increased intracellular survival of recombinant *M. smegmantis* expressing PE_PGRS41 within macrophage. Thus, our findings revealed *M. tuberculosis* PE_PGRS41 alteres the immune environment of the host cells, which might be involved in the pathogenesis of mycobacterial disease and hence influenced host cell responses to *M. tuberculosis* infection.

## Results

### PE_PGRS41 affects the growth of *M. smegmatis* upon exposure to hygromycin

**Figure 1:**
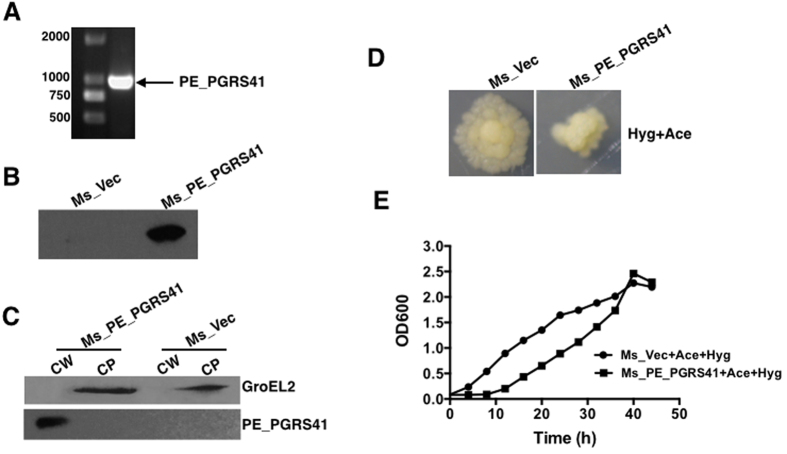
The effect of PE_PGRS41 on the growth of *M. smegmatis*. (**A**) PCR amplification of PE_PGRS41 encoding sequence from *M. tuberculosis* genome approximately 1086 bp. (**B**) *M. tuberculosis* PE_PGRS41 was expressed in *M. smegmatis* and detected using Western blotting. Cell lysates of Ms_Vec and Ms_PE_PGRS41 were subjected to Western blot to determine the expression of His-tagged PE_PGRS41 protein in *M. smegmatis* by anti-His antibody. (**C**) Cell fractionation experiments were performed to determine the sub-cellular localization of PE_PGRS41, GroEL2 protein serves as a cytoplasm marker of *M. smegmatis*, CW represents cell wall; CP represents cytoplasm. (**D**) The morphology of Ms_Vec and Ms_PE_PGRS41 were detected after induction by acetamide in the presence of hygromycin. (**E**) Ms_Vec and Ms_PE_PGRS41 were grown in Middlebrook 7H9 medium supplemented with 0.05% Tween 80, 1% acetamide and 0.2% glycerinum, with hygromycin (100 μg/ml). The OD_600_ was determined at an interval of 4 h.

To investigate the effect of PE_PGRS41 on the macrophage response to bacterial infection, we generated recombinant *M. smegmatis* strains. The PE_PGRS41 gene (1086 bp) was amplified from *M. tuberculosis* H37Rv genome using specifical primers (Table 1) and used to construct recombinant Ms_PE_PGRS41 (Fig. 1A). The Ms_PE_PGRS41 strain was engineered to express a His-tagged PE_PGRS41 protein from a recombinant PALACE vector, while the Ms_Vec strain harbored the vector alone. Both Ms_Vec and Ms_PE_PGRS41, which were grown in Middlebrook 7H9 medium in the presence of hygromycin (Hyg). PE_PGRS41 successfully expressed in *M. smegmatis* (Fig. 1B) and localized to cell wall component of *M. smegmatis* (Fig. 1C). To test the effect of PE_PGRS41 on recombinant strains, the growth rates of Ms_Vec and Ms_PE_PGRS41 were detected. No growth difference was detected in Ms_Vec and Ms_PE_PGRS41 strain with/without acetamide (Ace) or Hyg (Fig. 1S), demonstrating that the expression of PE_PGRS41 does not affect the growth of *M. smegmatis*. Notably, although both Ms_Vec and Ms_PE_PGRS41 habored the PALACE vector with anti-Hyg resistance gene, the cell size of recombinant Ms_PE_PGRS41 became smaller than Ms_Vec when exposure to Hyg after induction by Ace (Fig. 1D). Moreover, Hyg exposure delayed the growth of Ms_PE_PGRS41 strains while with negligible effect upon Ms_Vec after Ace induction (Fig. 1E). These data suggest that PE_PGRS41 might affect the growth of *M. smegmatis* upon exposure to antibiotic.

### PE_PGRS41 sensitizes *M. smegmatis* response to multiple antibiotics

**Figure 2:**
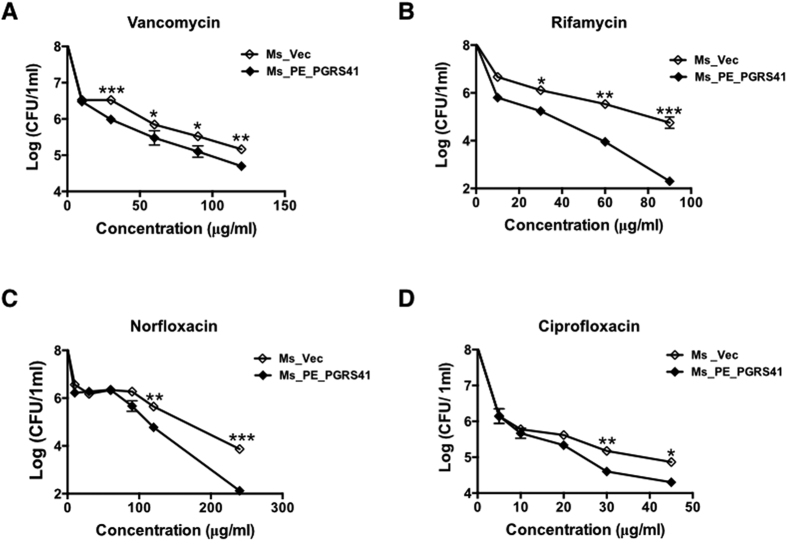
*M. tuberculosis* PE_PGRS41 expression in *M. smegmatis* decreased bacterial survival following exposure to various antibiotics, including vancomycin (**A**), norfloxacin (**B**), isoniazid (**C**), and ciprofloxacin (**D**). Ms_Vec and Ms_PE_PGRS41 were exposed to different concentration of vancomycin, norfloxacin, isoniazid, and ciprofloxacin for 5 h, then ten-fold dilution spotted the bacteria on MB 7H9 supplemented with 1% agar, the bacterial number of Ms_Vec and Ms_PE_PGRS41 were counted after 3 days cultivation.

*M. tuberculosis* cell wall serves as an effective permeable barrier of antibiotics^9^. PE_PGRS41 was a cell wall associated protein of mycobacteria (Fig. 1C). To study whether PE_PGRS41 functions in cell wall permeability, recombinant Ms_PE_PGRS41 and Ms_Vec were treated with ten anti-tuberculosis drugs as described in the method and the MIC of each antibiotic was detected (Table 2). Both Ms_PE_PGRS41 and Ms_Vec displayed comparable susceptibility to ofloxacin (Ofl), tetracycline (Tet), streptomycin (Str), isoniazid (INH) and chloramphenicol (Chl). The MIC values of Ofl, Tet, Str, INH and Chl for Ms_Vec and Ms_PE_PGRS41 were 2 μg/ml, 1 μg/ml, 0.125 μg/ml, 4 μg/ml and 32 μg/ml, respectively. However, Ms_PE_PGRS41 was susceptible to ciprofloxacin (Cip), rifampicin (Rif), especially vancomycin (Van) and norfloxacin (Nor). The MIC values of Cip and Rif for Ms_Vec were 1 μg/ml and 16 μg/ml, while 0.5 μg/ml and 8 μg/ml for Ms_PE_PGRS41, respectively. The MIC values of Ms_PE_PGRS41 for Van and Nor were 4 and 8 fold lower than Ms_Vec, respectively (Table 2 and Fig. 2). These results suggest a novel function of PE family protein PE_PGRS41 in the susceptibility to antibiotics.

### PE_PGRS41 alters the cell wall permeability of *M. smegmatis*

**Figure 3:**
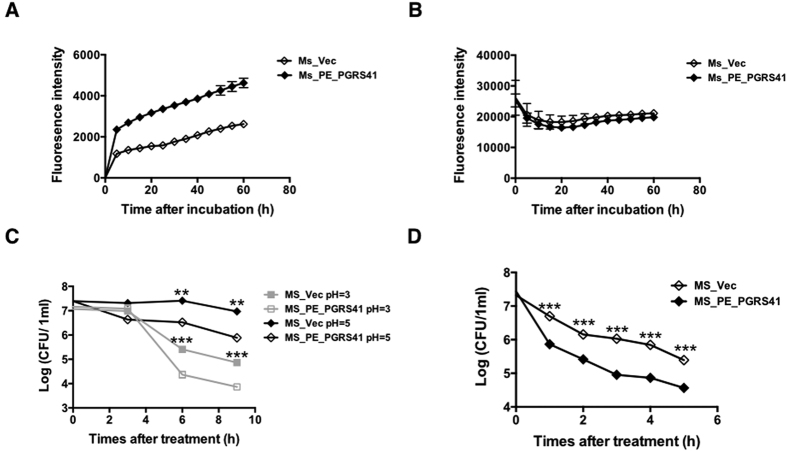
PE_PGRS41 expression in *M. smegmatis* results in increased cell wall permeability. (**A**) Mid-log-phase cultures of Ms_Vec and Ms_PE_PGRS41 were incubated in 7H9 containing 2 μg/ml ethidium bromide for indicated time. (**B**) Mid-log-phase cultures of Ms_Vec and Ms_PE_PGRS41 were incubated in 7H9 with 2 μM Nile red stain for indicated time. (**C**) The growth of Ms_Vec and Ms_PE_PGRS41 after treatment with different pH gradient for indicated time. The Ms_Vec and Ms_PE_PGRS41 strains were centrifuged, re-suspended to 5 ml MB 7H9 at an OD_600_ of 0.5, 10-fold serial dilutions of Ms_Vec and Ms_PE_PGRS41 were spotted on MB 7H10. (**D**) Mid-log-phase cultures of Ms_Vec and Ms_PE_PGRS41 were incubated in MB 7H9 supplemented with 0.05% SDS for indicated time. And then the recombinant strains were plated onto 7H10 plates by serially ten-fold dilution, the bacterial numbers were counted after 3–4 days of cultivation.

Recombinant Ms_PE_PGRS41 was more sensitive to multiple antibiotics, including norfloxacin, rifampicin, vancomycin and Ciprofloxacin. To examine whether cell permeability alteration plays a role in the increased sensitivity of PE_PGRS41 recombinant, we used fluorescence spectroscopy to measure the whole-well accumulations of Nile Red and ethidium bromide (EtBr), representatives of the hydrophobic and hydrophilic compounds, respectively^10^,^11^. The results showed that EtBr accumulated more rapidly and to higher levels in Ms_PE_PGRS41 than the Ms_Vec, indicating an increase in cell wall permeability (Fig. 3A), while no difference was detected in Nile red accumulation between above mentioned two recombinant strains (Fig. 3B). Consistent with a defective cell wall, Ms_PE_PGRS41 was more sensitive to different acid stress and the detergent sodium dodecyl sulphate (SDS) and acid stress. The survival ability of Ms_PE_PGRS41 was significant reduced by comparison to control strains Ms_Vec after 6 and 9 h treatment with acid stress (Fig. 3C). The survival of Ms_Vec was 10- to 100- fold increased in comparison to that of Ms_PE_PGRS41 when exposed to SDS for indicated time, suggesting defective cell wall integrality in Ms_PE_PGRS41 (Fig. 3D). These data suggest the PE_PGRS41 can alter the cell wall integrity and permeability of *M. smegmatis*.

### PE_PGRS41 enhances intracellular survival of *M. smegmatis* within macrophages

**Figure 4:**
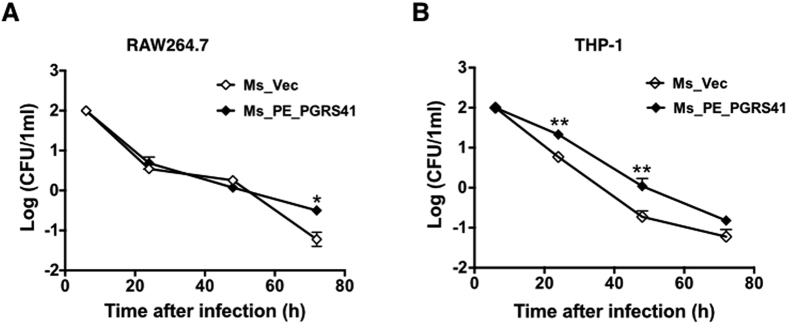
PE_PGRS41 increased the intracellular survival of *M. smegmatis*in macrophage after infection. (**A**) Mouse RAW264.7 cells were infected with Ms_Vec and Ms_PE_PGRS41 at an MOI of 10. (**B**) PMA-differentiated human THP-1 macrophages were infected with Ms_Vec or Ms_PE_PGRS41 at an MOI of 10. After 6, 24, 48, and 72 h infection, the macrophages were washed and lysed using 0.01% SDS. Lysates were plated on MB 7H10 medium to detect the bacterial number.

Several PE_PGRS proteins are responsible for the virulence *M. tuberculosis* and contribute to its ability to grow in a macrophage^12^,^13^,^14^. As the first step to determine the role of PE_PGRS41 in mycobacterial virulence, we performed macrophage infection experiments and compared the intracellular survival of the Ms_Vec and Ms_PE_PGRS41. PMA-differentiated THP-1 macrophages and murine RAW264.7 cells were infected with recombinant Ms_Vec and Ms_PE_PGRS41 strains at an MOI of 10 (10 bacterium for 1 macrophage cells) at 37 °C for 4 h, followed by washing and gentamicin treatment to remove extracellular bacteria. The intracellular growth of bacteria was assayed by enumerating the CFU at different time points post infection. Inside macrophages, Ms_PE_PGRS41 survived better than Ms_Vec during the course of infection in both RAW264.7 cells (Fig. 4A) and THP-1 macrophages (Fig. 4B). To confirm this phenotype is not because of difference in entry of macrophages, the entry of macrophages was measured at an MOI of 25 to allow a more accurate estimate of entry. Immediately following the infection (4 h at 37 °C), macrophage cells were washed three times to remove extracellular bacteria, and the CFU recovered from macrophages was enumerated. The result showed that the Ms_Vec appears to enter macrophages at a rate equivalent to the Ms_PE_PGRS41 (Fig. 2S). The data suggest that recombinant *M. smegmatis* strains expressing PE_PGRS41 enhances survival in both murine and human macrophage cell lines, indicating a potential role for PE_PGRS41 in bacterial persistence.

### PE_PGRS41 promotes the death of macrophage

**Figure 5:**
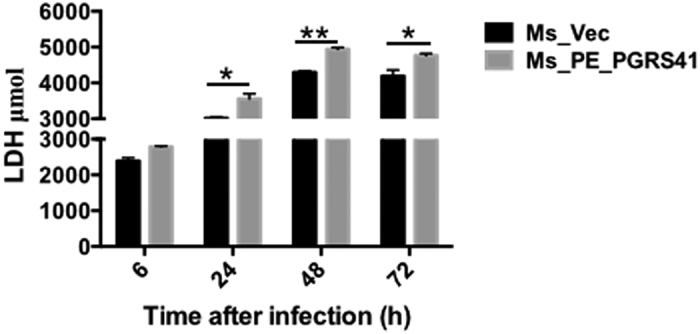
PE_PGRS41 promotes cell death of macrophage. Macrophages were infected with Ms_Vec or Ms_PE_PGRS41 at an MOI of 10. After 6, 24, 48, and 72 h infection, culture supernatants were collected and the release of LDH was measured. Data are shown as means ± SEM of triplicate wells.

Infection of macrophages with *M. tuberculosis* can induce necrosis, defined by cell lysis. One of the consequences of infection by *M. tuberculosis* is the induction of macrophage cell death either by apoptosis or by necrosis^15^, with different impact on the immunopathology of TB. Necrosis is believed to benefit the *M. tuberculosis* survival^16^. To determine whether necrosis of macrophage plays a role in PE_PGRS41 promoted intracellular survival, macrophages were infected with recombinant strains Ms_Vec or Ms_PE_PGRS41, LDH release in the culture supernatants was determined. LDH release from infected macrophage increased following 6 h infection with both Ms_PE_PGRS41 and the control strain Ms_Vec, Macrophages infected with Ms_PE_PGRS41 released more LDH compared to Ms_Vec after 24 h infection and later time points (Fig. 5), suggesting Ms_PE_PGRS41can precipitate the macrophage cell death.

### PE_PGRS41 inhibits macrophage pro-inflammatory cytokines production

**Figure 6:**
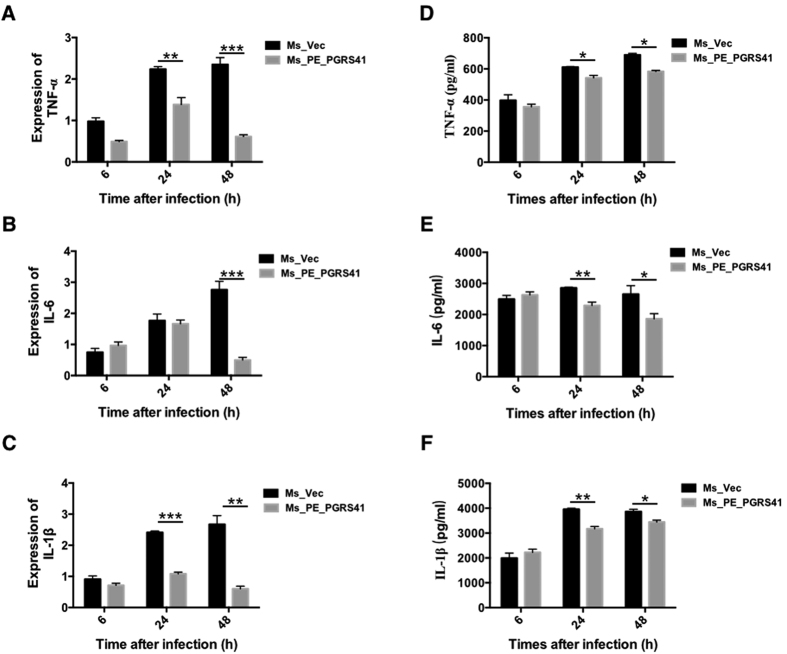
Overexpression of *M. tuberculosis* PE_PGRS41 in *M. smegmatis* diminishes the abundance of pro-informatory cytokines. PMA-differentiated THP-1 cells (2 × 10^6^/well/2 ml) were infected with recombinant Ms_Vec and Ms_PE_PGRS41 strains. After 6, 24, and 48 h infection, the infected cells and supernatant were collected. RT-PCR was used for analyze TNF-α mRNA (**A**), IL-6 mRNA (**B**), IL-1β mRNA (**C**). ELISA was used for detect the production of TNF-α (**D**), IL-6 (**E**), IL-1β (**F**).

Pro-inflammatory cytokines such as TNF-α, IL-6 and IL-1β are essential for the recruitment and activation of cells of the immune system in response to bacterial infection. To assess the immuno-modulatory potential of PE_PGRS41, we estimated the levels of several cytokines known to regulate the intracellular fate of *M. tuberculosis*^17^. We next investigated whether PE_PGRS41 modulates the expression of inflammatory cytokines in macrophages infected with Ms_Vec and Ms_PE_PGRS41. Expression of *M. tuberculosis* PE_PGRS41 in *M. smegmatis* inhibited the expression and production of pro-inflammatory cytokines TNF-α, IL-6 and IL-1β (Fig. 6), while the expression and secretion of anti-inflammatory cytokine IL-10 was increased in Ms_PE_PGRS41 infected macrophage in comparition to Ms_Vec infected macrophage (Fig. 3S). IL-10 inhibits the production of host-protective pro-inflammatory cytokines^18^ and is an inhibitor of early mycobacterial clearance^19^. We consistently observed a decrease in levels of TNF-α, IL-6 and IL-1β along with increased IL-10 transcript levels in infected macrophages. These data suggests that PE_PGRS41 plays a critical role in regulating cytokines expression.

### PE_PGRS41 suppresses the activation of macrophage

**Figure 7:**
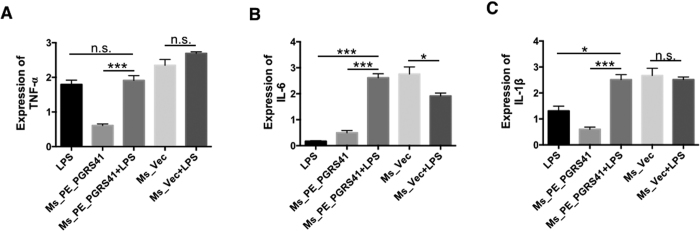
LPS priming recovers the cytokines production in Ms_PE_PGRS41 infected macrophages. LPS was added into culture 2 h prior to infection. The expression of TNF-α mRNA (**A**), IL-6 mRNA (**B**), and IL-1β mRNA (**C**) in THP-1 cells infected for 6, 24 and 48 h with *M. smegmatis* harbored PALACE (Ms_Vec) or PE_PGRS41-overexpressing *M. smegmatis* (Ms_PE_PGRS41).

PE_PGRS41 of *M. tuberculosis* was found to inhibit the transcription of pro-inflammatory cytokines such as TNF-α, IL-6 and IL-1β. Lipopolysaccharide (LPS), a structural component of the outer membrane of Gram-negative bacteria, is one of the most effective stimulators of the immune system^20^. To determine whether this phenotype is due to inhibited activation of macrophage, we treated THP-1 cells with LPS (100 ng/mL) and then determined whether PE_PGRS41 suppresses cytokines will be released from LPS-activated macrophage. We found LPS priming can recover the expression of the repressed the cytokines (TNF-α, IL-6 and IL-1β) after Ms_PE_PGRS41 infection (Fig. 6). When THP-1 cells were infected with Ms_PE_PGRS41 combined with a stimulatory dose of LPS, TNF-α (Fig. 7A), IL-6 (Fig. 7B) and IL-1β (Fig. 7C) expression were significantly increased compared to the response to LPS or Ms_PE_PGRS41 infection alone. This suggests that PE_PGRS41 can repress the activation of macrophage, resulting in decreased production of inflammatory cytokines in macrophages. Nitric oxide is potent antimicrobial agent acquired by innate cells and NO production is a critical defense mechanism in determining the outcome of TB infection, since it reduces the survival of *M. tuberculosis*^21^. To identify factors contributing to the increased intra-macrophage survival of the recombinant *M. smegmatis* strains, we measured the levels of inducible nitric oxide synthase 2 (iNOS), which is a key determinant of intracellular bacillary burden in host cells^22^,^23^. The expression level of iNOS in Ms_PE_PGRS41 infected THP-1 cells was detected by Western blotting, and the level of NO was measured by Griess reagent method. We observed comparable iNOS expression in Ms_Vec and Ms_PE_PGRS41 infected THP-1 macrophages after 6, 24, 48 and 72 h infection (Fig. 4S). There was no significant difference for the NO production between the PE_PGRS41 overexpression *M. smegmatis* and control (Fig. 4S).

### PE_PGRS41 represses cell apoptosis of macrophage

**Figure 8:**
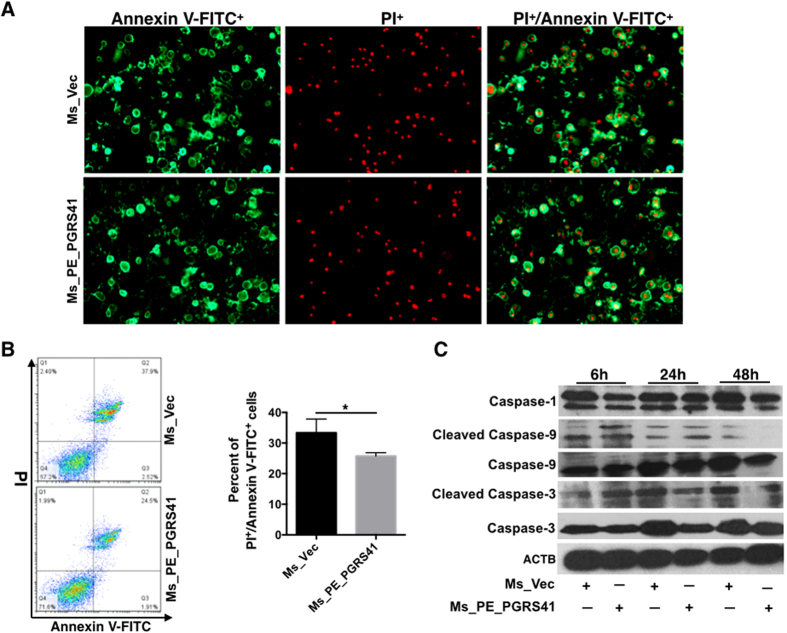
PE_PGRS41 depresses cell apoptosis of macrophage. Macrophages were infected with Ms_Vec and Ms_PE_PGRS41 for indicated time points, cells were collected and the level of apoptosis was detected by fluorescence microscope (**A**) and flow cytometry (**B**) through annexin-V-FITC and propidium iodide (PI) straining. (**C**) The expression of caspase-1, pro-caspase-3, pro-caspase-9, cleaved caspase-3 and caspase-9 were detected by Western Blot.

TNF-α and IL-1β provides protection against *M. tuberculosis* infection perhaps through the induction of host cell apoptosis^24^,^25^,^26^,^27^. Because we observed decreased transcript levels and production of TNF-α and IL-1β in Ms_PE_PGRS41 infected macrophages, we examined the hallmarks of apoptosis using fluorescence microscopy and Flow cytometry. The infected THP-1 cells stained with the Annexin V and PI that detects phosphatidylserine exposure on the outer leaflet of the plasma membrane and late apoptotic cells. Results showed that PE_PGRS41 overexpression markedly decreases the *M. smegmatis* infection-induced apoptosis of macrophage THP-1 cells when compared with Ms_Vec infected THP-1 cells (Fig. 8A). Flow cytometry validated this result (Fig. 8B). Several PE proteins-induced apoptosis has been shown to be dependent on activation of the caspase cascade with cleavage of caspases 3^28^. Moreover, the cleavage caspase-3 and caspase-9 were decreased in MS_PE_PGRS41 infected macrophage in comparion to Ms_Vec infected macrophage (Fig. 8C). These results may provides us that PE_PGRS41 inhibits cell apoptosis depends on caspase cascade.

### PE_PGRS41 suppresses the Autophagy via interfering ATG-8

**Figure 9:**
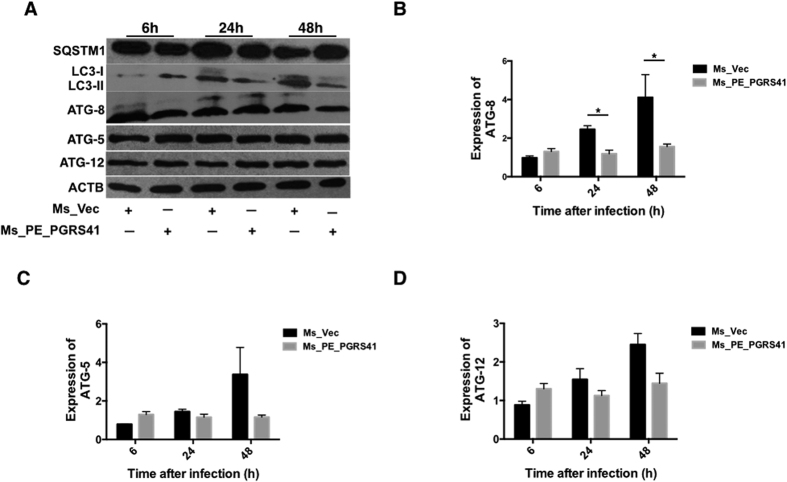
PE_PGRS41 suppresses autophagy of macrophage through interferon with ATG-8. PMA differentiated THP-1 cells were infected with Ms_Vec and Ms_PE_PGRS41 for indicated time. The cells were washed, collected and subjected to Western Blot for detection the expression of autophagy related protein (**A**). The expression of ATG-8 (**B**), ATG-5(**C**) and ATG-12 (**D**) mRNA was analyzed by RT-PCR.

Autophagy is intensely studied to test its role in host defense against *M. tuberculosis*^29^. We interested with if PE_PGRS41 can disturb autophagy. LC3 is a defined marker for autophagy, which plays an essential role in autophagosome formation. We measured LC3 protein expression in THP-1 cells infected with Ms_Vec or Ms_PE_PGRS41 and found that PE_PGRS41 blocks the expression level of LC3B (Fig. 9A). Ms_PE_PGRS41 infected macrophage suppressed the conversion of LC3-I to LC3-II. Hence, the relative ratio of LC3-II to LC3-I was substantially lower in Ms_PE_PGRS41 infected cells than Ms_Vec infected cells (Fig. 9A). Next, the SQSTM1/p62 protein serves as a marker to monitor autophagic flux. SQSTM1 is an ubiquitin-binding scaffold protein that is degraded by autophagy. Thus, decreased levels of SQSTM1 are observed when autophagy is activated^30^, whereas SQSTM1 accumulates when autophagy is inhibited^31^. Here, the SQSTM1 protein was detected in Ms_Vec and Ms_PE_PGRS41 infected cells; we found the expression of SQSTM1 significantly increased in cells infected with Ms_PE_PGRS41 by comparison to Ms_Vec infected cells (Fig. 9A). In addition, the expression of ATG8 protein (Fig. 9A) and ATG8 mRNA (Fig. 9B) were both inhibited after Ms_PE_PGRS41 infection, whereas no difference in other autophagy-related proteins such as ATG-5 (Fig. 9A and C) and ATG-12 (Fig. 9A and D), as well as other autophagy related DRAM2H, ATG-10 and ATG-4c (Fig. 5S).

## Discussion

Deciphering the genome of *M. tuberculosis* reveals a special PE family protein, which accounts for about 4% of whole genome, with a total of 99 members^4^. Based on whether C-terminal of PE-encoding proteins bearing Gly-Gly-Ala or Gly-GlyAsn multiple tandem repeat structure, it is divided into two subfamilies: PE_ and PE_PGRS. N-terminal PE domain contains highly conserved amino acid residues, C-terminal PGRS domain characterizes polymorphic GC-rich sequence, which varies in sequence, size and repeat numbers^32^. The existence and expansion of PE_PGRS genes in certain mycobacteria are associated with the acquisition of new virulence traits^3^,^33^. PE_PGRS proteins are major source of antigenic variation^6^, function as lipase to supply energy for *M. tuberculosis* persistence and survival^34^, decrease cytokine production and inhibit phagolysosome maturation of macrophages^35^,^36^. The role of most PE_PGRS proteins in *M. tuberculosis* biology and pathogenesis remains elusive despite sporadic studies^33^,^37^.

In this study, we found heterologous expression of *M. tuberculosis* PE_PGRS41 in *M. smegmatis* becomes extremely sensitive to multiple antibiotics and *in vitro* stresses through increasing the cell wall permeability (Figs 2 and 3). *M. smegmatis* expressing PE_PGRS41 showed enhanced survival both in the RAW264.7 and THP-1 macrophages (Fig. 4). The increased survival within the macrophage cannot be attributed to the increased resistance to any of the tested stresses^38^, as PE_PGRS41 promoted *M. smegmatis* is more sensitive to *in vitro* stress (Fig. 3). Recombinant Ms_PE_PGRS41 suppressed up to ~80% of the cytokine expression at various time points, starting from 24 h after infection, although survival of *M. smegmatis* did not change substantially until very late time points after infection in murine RAW264.7 macrophage (Fig. 4). Since Ms_Vec and Ms_PE_PGRS41 showed the same growth kinetics (Fig. 1C), the enhanced intracellular survival in macrophages of Ms_PE_PGRS41 might be the effects of PE_PGRS41 on innate immune response.

Immunoregulatory molecules, such as TNF-α^39^, IL-6^40^ and IL-1β^41^, are essential components of the host immune defense against mycobacterial infection^42^. *M. tuberculosis* effectors can suppress the expression of pro-inflammatory cytokines to benefit its survival in macrophages^36^,^43^,^44^. *M. tuberculosis* Rv0198c (zmp1), required for full virulence of the pathogen in mice, can suppress the IL-1β production by inhibiting inflammasome activation in murine macrophages^16^. Expression of PE_PGRS62 in *M. smegmatis* decreases pro-inflammatory cytokines IL-1β and IL-6 mRNA expression in macrophages after infection^36^. *M. tuberculosis* Mce3E inhibits TNF-α and IL-6 expression, and the promotion of mycobacterial survival within macrophages^43^. Consistent with that, we identified that *M. tuberculosis* PE_PGRS effector PE_PGRS41 down-regulates a specific pool of pro-immunoregulatory molecules, including TNF-α, IL-6 and IL-1β (Fig. 6), while the expression of anti-inflammatory factor IL-10 was increased in Ms_PE_PGRS41 infected macrophage in comparison to Ms_Vec infected cells (Fig. 3S). Studies have shown that IL-10 blocks phagosome maturation in M. tuberculosis infected human macrophages, resulting in the decreasing in pro-inflammatory cytokines production^45^,^46^. Thus, *M. tuberculosis* PE_PGRS41 has the ability to inhibit pro-inflammatory cytokines, resulting in the enhanced intracellular survival of mycobacteria in macrophages.

Recombinant *M. smegmatis* expressing PE_PGRS41 shows enhanced survival in both murine and human macrophage cell lines indicating a potential role for these proteins in bacterial persistence (Fig. 4). The association of PE_PGRS41 with the *M. smegmatis* cell wall (Fig. 1C) suggested a role in the mysterious mycobacterium-host interaction^47^,^48^. *M. tuberculosis* can interfere with a number of cellular processes critical for *M. tuberculosis* intracellular survival, such as phagosome maturation^49^, phagosome–lysosome fusion, phagosome acidification, apoptosis of infected macrophages^50^ and induction of autophagy^51^. PE_PGRS41 suppressed cytokines expression via blocking phagosome maturation, as LPS priming facilitated the expression of these cytokines after infection (Fig. 7).

Apoptosis is a host defence strategy used by the host to eliminate the intracellular bacteria^52^. We found that *M. smegmatis* PE_PGRS41 recombinant, instead of the control, inhibits macrophage apoptosis (Fig. 8) via casapse-dependant pathway. Autophagy is another host defence mechanism connected with apoptosis through several possible pathways^53^. Autophagy involves the formation of a double-membrane cytosolic vesicle, an autophagosome^54^,^55^. Central to the formation of the autophagosome is the ubiquitin-like protein autophagy-related LC-3. We found PE_PGRS41 suppresses the conversion of LC3-I to LC3-II, whereas significantly activated the expression of autophagic flux marker SQSTM1/p62 protein (Fig. 9). In addition, ATG-8 controls the expansion of the autophagosome precursor and determines the size of autophagosomes. We demonstrated that PE_PGRS41 expressing *M. smegmatis* blocks the ATG-8 protein and ATG-8 mRNA expression. Taken together, these data suggest that PE_PGRS41 may suppress autophagy via interaction with autophagy-related ATG-8.

In summary, our results defined a novel role of PE_PGRS41. PE_PGRS41 can decrease pro-inflammatory cytokines expression via blocking phagosome maturation, decreasing apoptosis and autophagy, resulting in the persistence in macrophage. PE_PGRS41 protein seems to be the sole factor mediating abovementioned effect. Hence, PE_PGRS41 represents an important mycobacterial virulence factor in the ongoing battle between host and pathogen.

## Methods

### Reagents

Middlebrook 7H10 agar and Middlebrook 7H9 broth medium were purchased from BD Difco Laboratories. Annexin V-FITC/PI detection kit was obtained from BD. Anti-caspase-1, anti-caspase-3, anti-caspase-9 (human specific), anti-LC3B, anti-SQSTM/p62, anti-ATG5, anti-ATG12, anti-ATG8 and anti-β-actin antibody were obtained from Cell Signaling Technology. PMA was obtained from Sigma–Aldrich (Sigma), dissolved in DMSO and stored at −20 °C. The final concentration of DMSO in all experiments was less than 0.1% trypsase. pALACE plasmid was a gift from professor Yossef Av-Gay (University of British Columbia).

### Bacterial and cell culture

#### Bacterial culture

*M. smegmatis* mc^2^155 and recombinant strains Ms_Vec and Ms_PE_PGRS41 were grown in Middlebrook 7H9 broth medium with or without hygromycin (Hyg), supplemented with 0.5% glycerol at 37 °C, 120 r.p.m. Recombinant strains Ms_Vec and Ms_PE_PGRS41 were grown in 7H9 medium containing 0.05% Tween 80,0.5% glycerol until logarithmic growth phase, the cultures were reinoculated in fresh 7H9 medium supplement with and 1% acetamide at the ratio of 1:1000 dilutions. Cultures were incubated at 37 °C with shaking through the entire growth phase. Samples were collected and the OD_600_ values were measured at every 4 h interval, the average values were used to plot growth curves.

#### Cell culture

THP-1 and RAW264.7 cells were cultured in RPMI 1640 supplemented with 10% fetal bovine serum (FBS) and 100 U/ml penicillin, 100 mg/ml streptomycin (GIBCO, Invitrogen), at 37 °C with an atmosphere of 5% CO_2_.

### Construct the recombinant strains

The intact PE_PGRS41 gene was amplified from *M.tuberculosis* H37Rv genome using gene-specific primers (forward with *Bam*H I site–5′-CGGGATCCATGTCGTTCCTGATTGCTT-3′; reverse with *Cla*I site-5′-CCATCGATTCGACGTGATCCAGA-3′ (Table 1). The amplified PE_PGRS41 gene was cloned into PGM@19-T vector (Promega, Madison, WI). The *Bam*H I-ClaI digested PCR product was then cloned to plasmid pALACE-His (vector) to generate the plasmid PALACE-PE_PGRS41. The plasmids (PALACE and PALACE-PE_PGRS41) were electroporated into *M. smegmatis*mc^*2*^155, a fast-growing non-pathogenic mycobacteria^56^. The electroporated recombinant *M. smegmatis* strains were plated on Middlebrook (MB) 7H10 agar containing 100 μg/ml hygromycin after *in vitro* growth in MB 7H9 liquid medium for 3 h. The constructs harboring PE_PGRS41 gene (Ms_PE_PGRS41) were confirmed by PCR amplification, and the positive recombinant strains were stored with sterile 20% glycerol at −80 °C for further use. *Escherichia coli* DH5α strains using for gene cloning were grown at 37 °C using Luria–Bertani (LB) broth and LB agar with the addition of appropriate antibiotics. *M. smegmatis* mc^2^155 were grown in 7H9 broth medium or on 7H10 agar supplemented with 0.05% (v/v) Tween 80, 0.2% (w/v) glucose and 0.5% (v/v) glycerol.

### Detection the expression of PE_PGRS 41 in *M. smegmatis*

The recombinant *M. smegmatis* strains Ms_Vec and Ms_PE_PGRS41 were cultured in 7H9 broth medium until the OD_600_ value between 0.6 and 0.8. Protein expression was induced using 1% (v/v) acetamide (Aladdin, China). *M. smegmatis* cell fractionation was carried out essentially as described earlier, with minor modifications^57^. In general, the recombinants including Ms_Vec and Ms_PE_PGRS41were harvested after 16 h acetamide induction using centrifugation at the speed of 3000 × g for 10 min at 4 °C. The collected cells were washed using cold PBS (phosphate-buffered saline). And then sonication was performed in cold PBS supplemented with protease inhibitor P-8849 (Sigma-Aldrich). After sonication, the prepared whole-cell lysate were centrifuged at the speed of 20000 × g for separating the insoluble (pellets in the bottom) and the soluble (supernatant in the upper layer) fractions. The separated fractions were loaded to SDS-PAGE and further detected by Western blot analysis using specific anti-His monoclonal antibody (TIANGEN, China). The blots were formed when incubated with IgG-HRP, anti-mouse IgG antibody labeled with horseradish peroxidase (TIANGEN, China).

### Localization of the PE_PGRS41 protein

Recombinant Ms_Vec and Ms_PE_PGRS41 constructs were grown and subjected to cell fractionation using the method previously described. Generally, the acetamide-induced recombinant Ms_Vec and Ms_PE_PGRS41 were subjected to sonication. The whole lysates were centrifuged at the speed of 3,000 × g for 5 min at 4 °C to remove un-lysed cells and cell debris. The supernatants were ultra-centrifuged at the speed of 27,000 × g for 30 min at 4 °C. After ultra-centrifugation, the pellets (cell wall) and the supernatants (cell membrane and cytosol fractions) were collected separately. The pellets were further suspended in PBS. Equal amounts of protein from pellets and supernatants fraction were subjected to Western blotting to determine the expression of PE_PGRS41, using cytosol marker protein GroEL2 as control.

### *In vitro* survival under different stresses

To examine their growth patterns, recombinant *M. smegmatis* strains were cultured in 7H9 medium containing 100 μg/ml hygromycin and grown into an optical density (OD_600_) of 0.8. The expression of PE_PGRS41 was induced by acetamide. OD plotted growth curves against culture time. Acetamide induced Ms_Vec and Ms_PE_PGRS41 were performed in the presence of surface stress, and acidic pH stress. For surface stress, 0.05% SDS was treated for 1, 2, 3 and 4 h. For acidic pH stress, pH gradient was generated by adding HCl in 7H9 medium and was adjusted down to 3 and 5 filter sterilized by passing through a 2 μm filter. After the treatment with SDS and acid stress, the recombinant strains were diluted and plated into MB 7H10 agar containing hygromycin for bacteria enumeration.

### Anti-tuberculosis drug sensitivity assays

Ten antibiotics were used in this study. Acetamide induced Ms_Vec and Ms_PE_PGRS41 strains were prepared for treatment with the ten antibiotics. MIC values of each antibiotic were determined when the bacterial activity was killed at least 99% on liquid medium. After 24 h treatment with these ten antibiotics with different concentration, the bacteria were diluted by 10-fold and plated into 7H10 agar medium and counted the bacterial number after 3 days culture. The medium without any antibiotics serves as the control to confirm the normal growth of bacteria.

### *In vitro* infection with recombinant *M. smegmatis*

THP-1, the suspension cell line was cultured in RPMI 1640 medium (Invitrogen) supplemented with 10% (v/v) heat inactivated FBS, 2 mM L-glutamine, 100 μg/ml streptomycin and 100 U/ml penicillin (Invitrogen) and cultured in humidified incubator supplemented with 5% CO_2_ at in 37 °C. Cells were seeded at 2 × 10^6^ cells/well in 6-well tissue culture plates and differentiated by stimulation with 100 ng/ml PMA. The differentiated THP-1 cells were washed and changed the culture without any antibiotics, and then the cells were infected with Ms_Vec and Ms_PE_PGRS41at an MOI of 10. Four hours after infection, the infected cells were washed using PBS and gentamicin (100 μg/ml) was used to kill the bacteria outside the macrophages after four hours infection. For lactate dehydrogenase (LDH) activity assay, the culture supernatants were collected from infected cells after 6, 24, 48 h and 72 h infection. The LDH activities were detected using commercially LDH cytotoxicity kit (Takara Bio) according to standard procedure. For bacteria survival within macrophage, THP-1 cells were infected with Ms_Vec and Ms_PEPGRS41 at an MOI of 10 for 6 h, 24 h, 48 h and 72 h at 37 °C. Then the infected macrophages were washed 3 times using PBS and lysed in 1 ml 0.025% SDS. The cell lysates were diluted and aliquots of each dilution were plated on 7H10 agar plates supplemented with 10% glycerol. After 3 days incubation, colonies on plates were counted. The survival rate was calculated as compared to the control.

### RT-PCR and Assay for cytokines

PMA-differentiated THP-1 cells were infected with Ms_Vec and Ms_PEPGRS41 at an MOI of 10. After 6, 24 and 48 h infection, total cellular RNA was extracted from cells was extracted from the infected cells using RNA extraction kit (TIANGEN) according to the manufacturer’s recommendations. cDNA synthesis was performed using the PrimeScript RT reagent kit (Takara, Shiga, Japan). Quantitative real-time RT-PCR reactions were performed using a CFX96 RT-PCR Detection System (Bio-Rad) using SYBR Green Master Mix. Relative mRNA levels were calculated after normalizing to β-actin. The primers of listed in Table 1. Culture supernatants were collected from the infected macrophages, the cytokines production were detected with commercialy available ELISA kits for TNF-α, IL-6, IL-1β and IL-10 (eBioscience).

### Apoptosis analysis

2 × 10^6^ THP-1 cells were infected with Ms_Vec and Ms_PE_PGRS41 for 6 h and 48 h, the infected cells were washed with ice-cold PBS and the apoptotic cells were determined by Annexin V-FITC and propidium iodide (PI) straining according to manufacturer’s instructions (Beibo, Shanghai, China). This product detects the externalization of phosphatidylserine in apoptotic cells using recombinant annexin V conjugated to green-fluorescent FITC dye and dead cells using propidium iodide (PI). The cells were subjected to fluorescence microscope analysis and flow cytometer. Untreated cells were taken as negative control.

### Detection of NO production

THP-1 cells were infected with Ms_PE_PGRS41 vs. control Ms_Vec, the supernatant of each sample was collected at 6, 24, 48 and 72 h after challenge. NO levels were determined by measuring its stable end product, nitrite, using a Griess reagent (Jiancheng Bioengineering Institute, Nanjing). One hundred microliter of supernatant was added to an equal volume of Griess reagent in duplicate on a 96-well plate and incubated at room temperature for 15 min. Absorbance (540 nm) was measured and nitrite concentrations were estimated using a standard nitrite curve. Results were expressed as the mean micromoles of nitrite per sample ± SEM.

### Western blot

Western blot was performed as described previously. Cells were washed three times with ice-cold PBS and then lysed in lysis buffer containing 1 mM phenylmethylsulfonyl fluoride, 1% (v/v) protease inhibitor cocktail (Sigma). Lysates were centrifuged at 12,000 × *g* for 15 min, and the protein concentration was measured using a bicinchoninic acid (BCA) Protein Assay kit (TIANGEN Biotechnology, China). Equal amounts of cell lysates were separated by SDS-PAGE and then transferred to nitrocellulose membranes. Membranes were blocked in 5% non-fat dry milk in TBST and incubated overnight with the respective primary antibodies against caspase-1, caspase-3, caspase-9, LC3B, SQSTM/p62, ATG-5, ATG-12, ATG-8 and His-tag (dilution 1: 1000). β-actin serves as an internal control. The membranes were incubated at room temperature for 1 h with appropriate HRP-conjugated secondary antibodies and X-ray film was developed using Plus-ECL chemiluminescent reagent.

### Statistical analysis

Data were expressed as the mean ± SEM of at least three independent experiments. Statistical analysis was performed using GraphPad Prism 6.0. The results from RT-PCR, Griess assay and CFU assay were compared by Student’s t test. Differences were considered statistically significant with *P < 0.05, **P < 0.01, and ***P < 0.001.

## Additional Information

**How to cite this article:** Deng, W. *et al. Mycobacterium tuberculosis* PE_PGRS41 Enhances the Intracellular Survival of *M. smegmatis* within Macrophages Via Blocking Innate Immunity and Inhibition of Host Defense. *Sci. Rep.* **7**, 46716; doi: 10.1038/srep46716 (2017).

**Publisher's note:** Springer Nature remains neutral with regard to jurisdictional claims in published maps and institutional affiliations.

## Supplementary Material

Supplementary Material

## Figures and Tables

**Table 1:** Primers used in this study.

Primer	Sequence (5′–3′)
IL-1β (F)	CTGAAGGCCCGAATGCACCAG
IL-1β (R)	GCAAAGGTGGTGTCAGTATC
TNF-α (F)	GTAATGCTCCTCCCTACTTC
TNF-α (R)	GCAAAGGTGGTGTCAGTATC
IL-6 (F)	TCTCTGGTGACATGAAGAAGCT
IL-6 (R)	GCAAAGGTGGTGTCAGTATC
IL-10 (F)	AGACTCTGCTTCCTGATTGG
IL-10 (R)	GTGAGTGTCCCTGCTGGTC
ATG-5 (F)	AAGCAACTCTGGATGGGATTG
ATG-5 (R)	CAGGATCAATAGCAGAAGGAC
ATG-10 (F)	CCCAGCAGGAACATCCAATA
ATG-10 (R)	AGGCTCAGCCATGATGTGAT
ATG-12 (F)	AGTAGAGCGAACACGAACCATCC
ATG-12 (R)	AAGGAGCAAAGGACTGATTCACATAA
ATG-4c (F)	CAGCTGTGGTTGCTCACATTT
ATG-4c (R)	CTAAGTAGTCGGTGTTGGTTC
ATG-8 (F)	TCGGAGAAGACCTTCAAG
ATG-8 (R)	CATGTTGACATGGTCAGG
DRAM2H (F)	AAGCAAGTTCATGCTCTGATC
DRAM2H (R)	CCAGATAACCAACAACAGTCTG
β-actin (F)	TTCCTTCCTGGGCATGGAGTCC
β-actin (R)	TGGCGTACAGGTCTTTGCGG

**Table 2:** The MIC of recombinant Ms_Vec and Ms_PE_PGRS41 to different antibiotics.

Antibiotics (ug/ml)	Ms_Vec	Ms_PE_PGRS41
Isoniazid (INH)	4	4
Norfloxacin (NOR)	16	2
Ciprofloxacin (Cip)	1	0.5
Rifampicin (Rif)	16	8
Gentamicin (Gen)	1	0.5
Tetracycline (Tet)	1	1
Ofloxacin (Ofl)	2	2
Vancomycin (Van)	5	1.25
Streptomycin (Str)	0.125	0.125
Chloramphenicol (Chl)	32	32
