# Supplementary material for: Mycobacterium tuberculosis PE_PGRS41 Enhances the Intracellular Survival of M. smegmatis within Macrophages Via Blocking Innate Immunity and Inhibition of Host Defense
Source: Sci Rep. 2017 Apr 25;7:46716. doi: 10.1038/srep46716 (PMC5404228; doi:10.1038/srep46716)
Supplement: Supplementary Material [file srep46716-s1.pdf]

Mycobacterium tuberculosis PE_PGRS41 Enhances the Intracellular Survival of *M. smegmatis* within Macrophages Via Blocking Innate Immunity and Inhibition of Host Defense

Wanyan Deng[1,2#], Quanxin Long[2#], Jie Zeng[1], Ping Li[1], Wenmin Yang[1], Xinchun Chen[3*] and JianpingXie[1*]

1 State Key Laboratory Breeding Base of Eco-Environment and Bio-Resource of the Three Gorges Area, Key Laboratory of Eco-environments in Three Gorges Reservoir Region, Ministry of Education, School of Life Sciences, Institute of Modern Biopharmaceuticals, Southwest University, Chongqing, China.

2. Key Laboratory of Molecular Biology for Infectious Diseases (Ministry of Education), Institute for Viral Hepatitis, Department of Infectious Diseases, The Second Affiliated Hospital, Chongqing Medical University, Chongqing, PR China.

3.Department of Pathogen Biology, Shenzhen University School of Medicine,3688, Naihai Bvld, Shenzhen 518060, China

# These authors contributed equally to this work, co-first author

*Correspondence to: Jianping Xie, email: georgex@swu.edu.cn, phone: +8602368253392. Xinchun Chen chenxinchun@szu.edu.cn

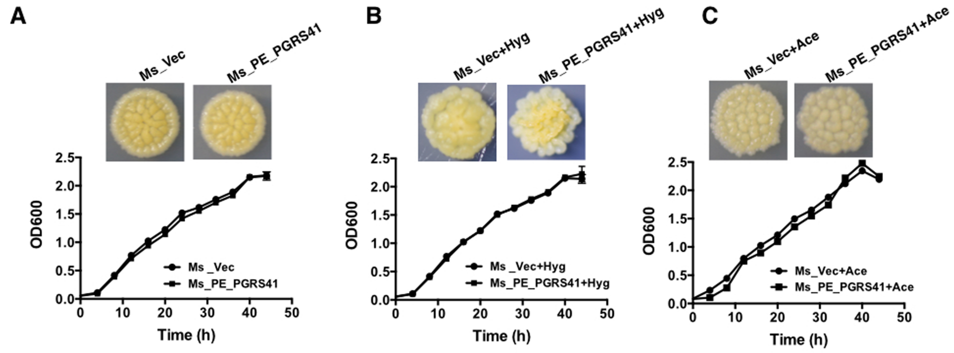

Fig. 1S. The effect of PE_PGRS41 on the morphology and growth of *M. smegmatis*. (A) Ms_Vec and Ms_PE_PGRS41 were grown in Middlebrook 7H9 medium supplemented with 0.05% Tween 80, and 0.2% glycerinum. (B) Ms_Vec and Ms_PE_PGRS41 were grown in Middlebrook 7H9 medium supplemented with 0.05% Tween 80, 0.2% glycerinum and hygromycin (100 μg/ml). (C) Ms_Vec and Ms_PE_PGRS41 were grown in Middlebrook 7H9 medium supplemented with 0.05% Tween 80, 0.2% glycerinum and 1% acetamide. The OD_{600} was determined at an interval of 4 h. The morphology of Ms_Vec and Ms_PE_PGRS41 from (A), (B) and (C) were detected, respectively.

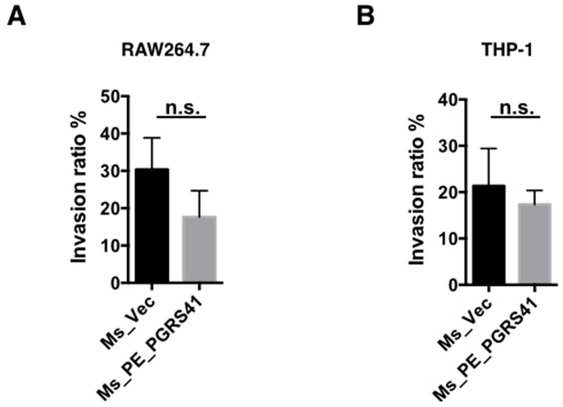

Fig. 2S. The equivalent entry ratio of recombinant strains to macrophages. Infection of both RAW264.1 cells (A) and THP-1 macrophages (B) with recombinant Ms_Vec and Ms_PE_PGRS41 at an MOI of 25, after 4h infection, the macrophages were washed and lysed using 0.01% SDS. Lysates were plated on MB 7H10 medium to detect the bacterial number. The bacterial numbers were counted after 3-4 days of cultivation at 37℃.

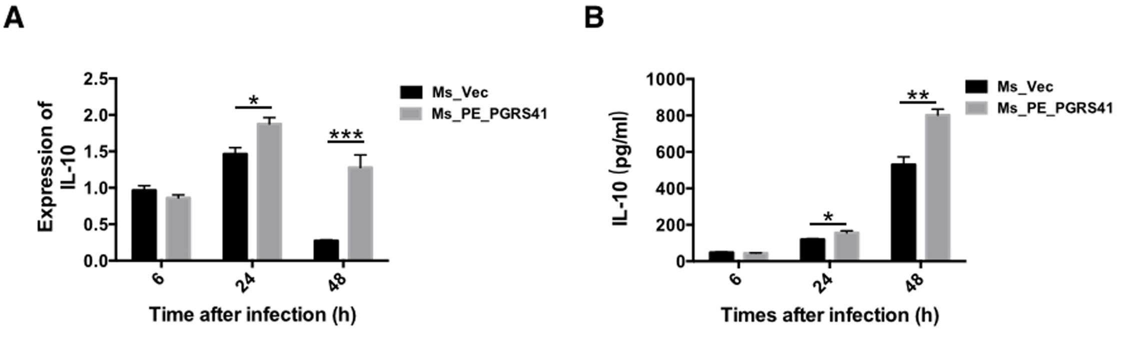

Fig. 3S. Expression of PE_PGRS41 in *M. smegmatis* promotes IL-10 production. PMA-differentiated THP-1 cells (2 × 10^6/well/2 ml) were infected with recombinant Ms_Vec and Ms_PE_PGRS41 strains for 6, 24, and 48 h, the infected cells were collected and subjected to RT-PCR analysis the expression of IL-10 mRNA. the infected cells and supernatant were collected. RT-PCR was used for analyze IL-10 mRNA (A) and ELISA was used for detect the production of IL-10 (B).

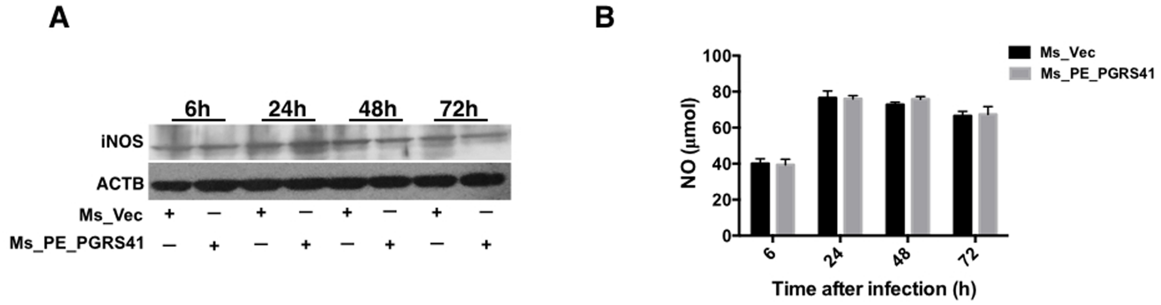

Fig. 4S. PE_PGRS41 has no effects on iNOS expression and NO production of macrophage. THP-1 cells were infected with Ms_Vec and Ms_PE_PGRS41 for 6, 24, 48 and 72h. The expression of iNOS (A) and the production of NO (B) were measured by Western Blotting and Griess assay, respectively.

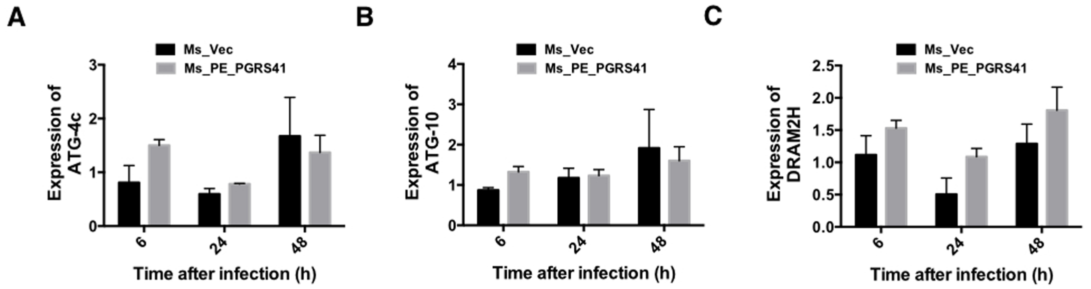

Fig. 5S. The expression of other autophagy-related genes after infection. Infection of macrophages with recombinant Ms_Vec or Ms_PE_PGRS41 at an MOI of 10 for 6, 24 and 48h, the infected cells were subject to RT-PCR to detect the expression of ATG-4c mRNA (A), ATG-10 mRNA (B), DRAM2H mRNA (C).
